# An Uncommon Deformity of the Collarless, Polished, Double-Taper (CPT) Stem After a Periprosthetic Fracture in Total Hip Arthroplasty

**DOI:** 10.7759/cureus.80197

**Published:** 2025-03-07

**Authors:** Julia E.J.W. Geilen, Isobel M Dorling, Bert Boonen, Thijs A. Nijenhuis

**Affiliations:** 1 Orthopaedic Surgery, Zuyderland Medical Centre, Sittard-Geleen, NLD; 2 Orthopaedics and Traumatology, SKB Winterswijk, Winterswijk, NLD; 3 Orthopaedics and Trauma, Zuyderland Medical Centre, Sittard-Geleen, NLD

**Keywords:** cpt stem, peri prosthetic femoral fracture, stem deformity, total hip arthroplasty: tha, trauma and ort

## Abstract

We present an uncommon case of a Collarless, Polished, Double-Taper (CPT) stem deformity (irreversible stem bending) occurring due to a Periprosthetic Fracture (PPF) in Total Hip Arthroplasty (THA). The entire course of the case, including treatment, risk factors, and failure analysis of the deformed CPT by its manufacturer, Zimmer Biomet (Warsaw, USA),is described. The discussion considers in detail the (risk) factors that led to this particular stem deformity, incorporating current literature. We conclude with learning points on how to prevent stem abnormalities in PPFs in the future.

In our case, there were some case-specific risk factors associated with bending of the CPT stem. These factors are a relative varus angle, relative under-sizing of the stem, and a potentially inadequate cement mantle around the primary stem. No material or manufacture failure of the stem could be found. Still, it is very unusual that the femoral stem deforms without fracturing. Taken together, we present a unique case of a Periprosthetic Fracture with CPT stem deformity following Total Hip Arthroplasty.

## Introduction

This case report describes an uncommon case of a Periprosthetic Fracture (PPF) in Total Hip Arthroplasty (THA). After primary THA the risk of PPF is estimated between 0.4% - 3.5% with a prevalence of less than 1%. These fractures are rare but feared and dreaded as a complication after THA. 

The cause is mostly due to a low-energy (minor) trauma, like in our case [1, 2]. Based on current literature, an even rarer form of PPF is the fracturing of the femoral stem by trauma [3, 4]. 

This case report presents a unique case of PPF. Due to the fracture, the implanted Collarless, Polished, Double-Taper (CPT) femoral stem was deformed (irreversibly bent) but not fractured. 

The orthopaedic experts involved in this uncommon case were interested in whether there were any explanations for this rare CPT stem bending. To the best of our knowledge, no bending of a femoral stem is described in the literature yet. A thorough case study of the patient’s medical record and the current literature was performed to indicate risk factors or explanations. Zimmer Biomet (Warsaw, USA), as the manufacturer of the CPT stem, investigated whether there were any manufacturer errors or abnormalities that contributed to this rare deformity.

We present a critical reflection on our orthopaedic interventions. The patient was followed carefully, up to six months postoperative, to make sure no additional, uncommon, complications occurred. 

The legal caregiver of the patient presented in this case was informed that data and radiographs would be submitted for publication. Written informed consent was obtained.

## Case presentation

A woman in her 60s was presented at the emergency department of a local hospital. She had a medical history of epilepsy and cognitive impairment due to a hemorrhagic stroke several years ago. Due to this, she resided in an assisted living facility. She did not take any medication. 

**Figure 1:**
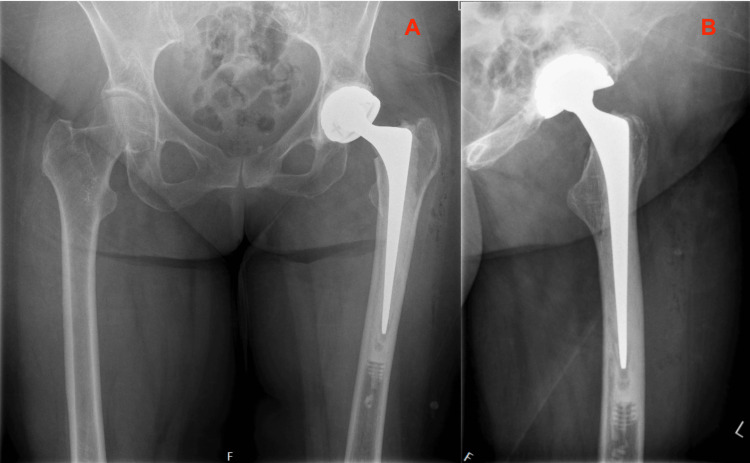
Primary Total Hip Arthroplasty Routine direct postoperative radiographs of the primary THA with CPT femoral stem, performed for a femoral neck fracture. 1A: Anterior Posterior view of the Pelvis; 1B: Lateral view of the Left Hip THA: Total Hip Arthroplasty; CPT: Collarless, Polished, Double-Taper

Three months prior she received a cemented (Optipac^©^ 80 Refobacin^©^ Bone Cement R by Zimmer Biomet, Warsaw, USA), dual mobility THA after a femoral neck fracture on the left side (Figure 1). 

**Figure 2:**
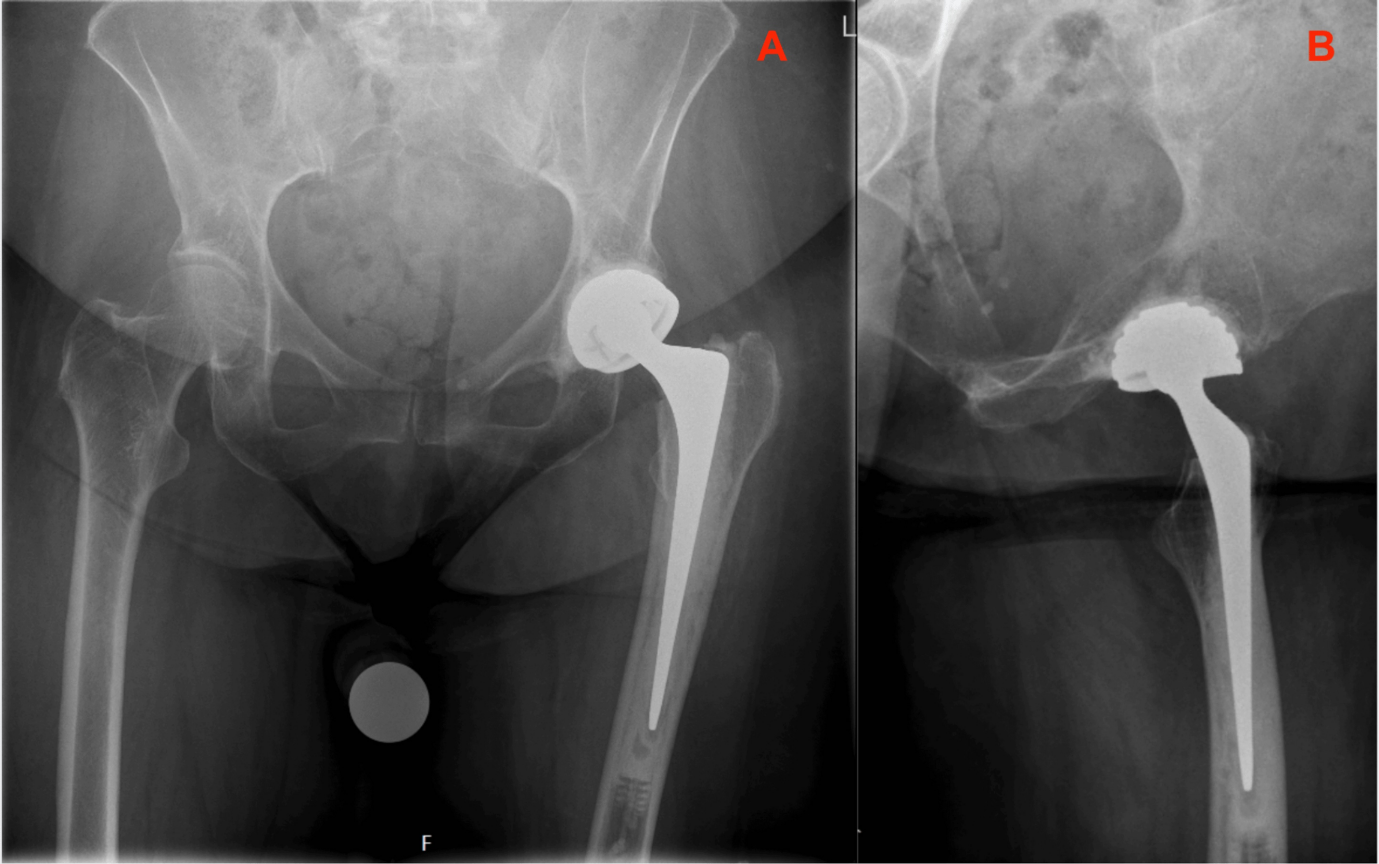
Primary Total Hip Arthroplasty, six weeks postoperative Six weeks postoperative radiographs of the primary THA with CPT femoral stem, performed for a femoral neck fracture. 2A: Anterior Posterior view of the Pelvis; 2B: Lateral view of the Left Hip THA: Total Hip Arthroplasty; CPT: Collarless, Polished, Double-Taper

Due to the cognitive impairment, her overall activity level was low and her risk of falling compared to healthy 60-year-olds was increased. Rehabilitation happened, however, without any problems. At the routine six-week follow-up the patient was independently mobile without needing walking aids. Radiographs of the primary THA were without signs of surgical complications (Figure 2).

**Figure 3:**
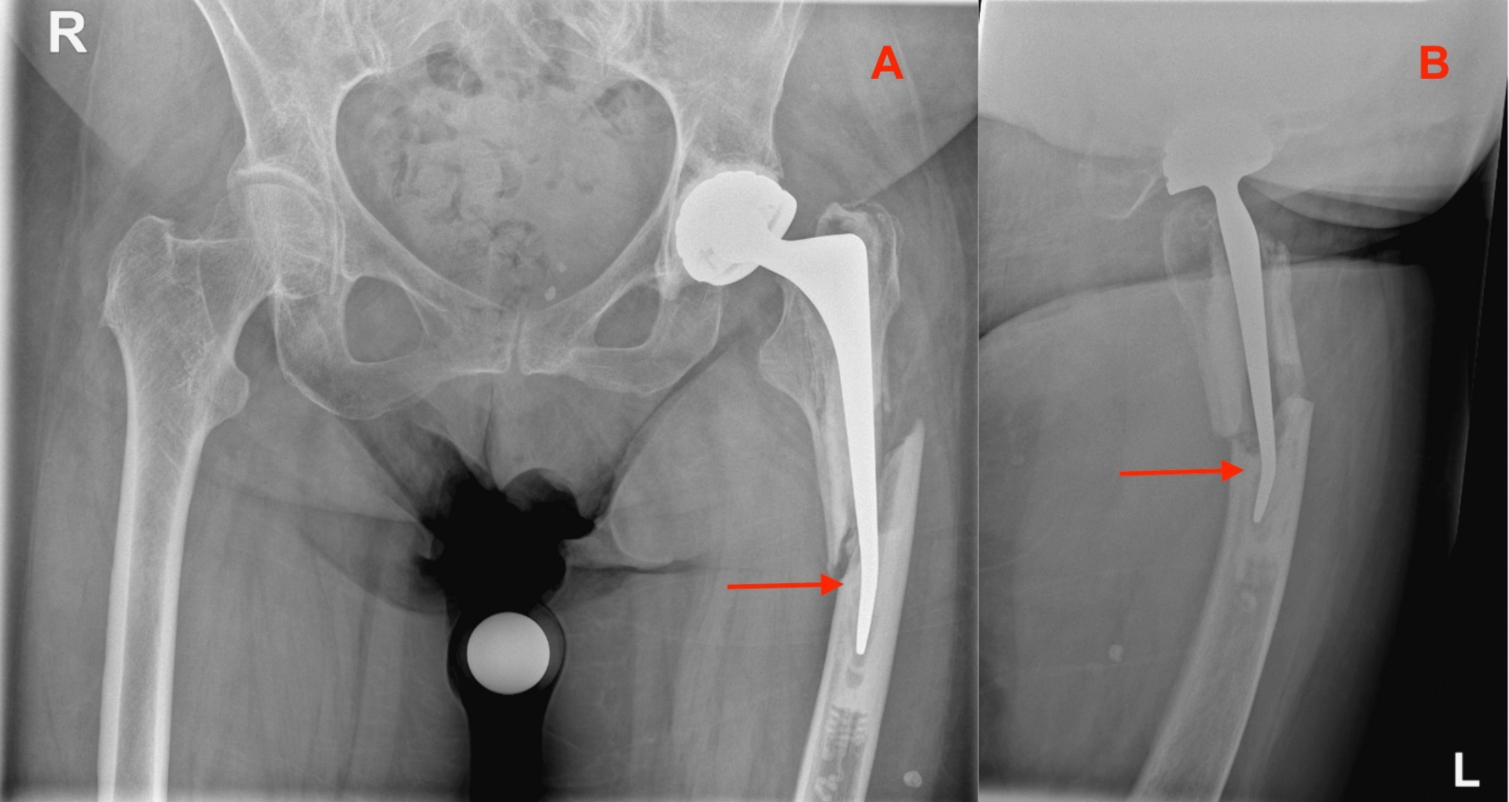
Vancouver B2 periprosthetic fracture with CPT stem bending (arrows) Radiographs of the pelvis and left hip performed at the emergency department where the patient was seen with complaints of left-sided hip pain. 3A: Anterior Posterior view of the Pelvis; 3B: Lateral view of the Left Hip CPT: Collarless, Polished, Double-Taper

The reason for evaluation in the emergency department was because the patient most likely fell on her left hip that morning. The exact trauma mechanism was not observed, therefore, the patient was fully screened to avoid overlooking any fractures. Clinical examination of the hip or standing on her left leg was not possible due to excessive pain. No hematomas or skin lacerations were visible in that area. Additionally, no neurovascular abnormalities of the left leg were found. Radiographic imaging of the pelvis and left hip showed a PPF of her cemented, dual mobility THA from three months before (Figure 3). 

**Figure 4:**
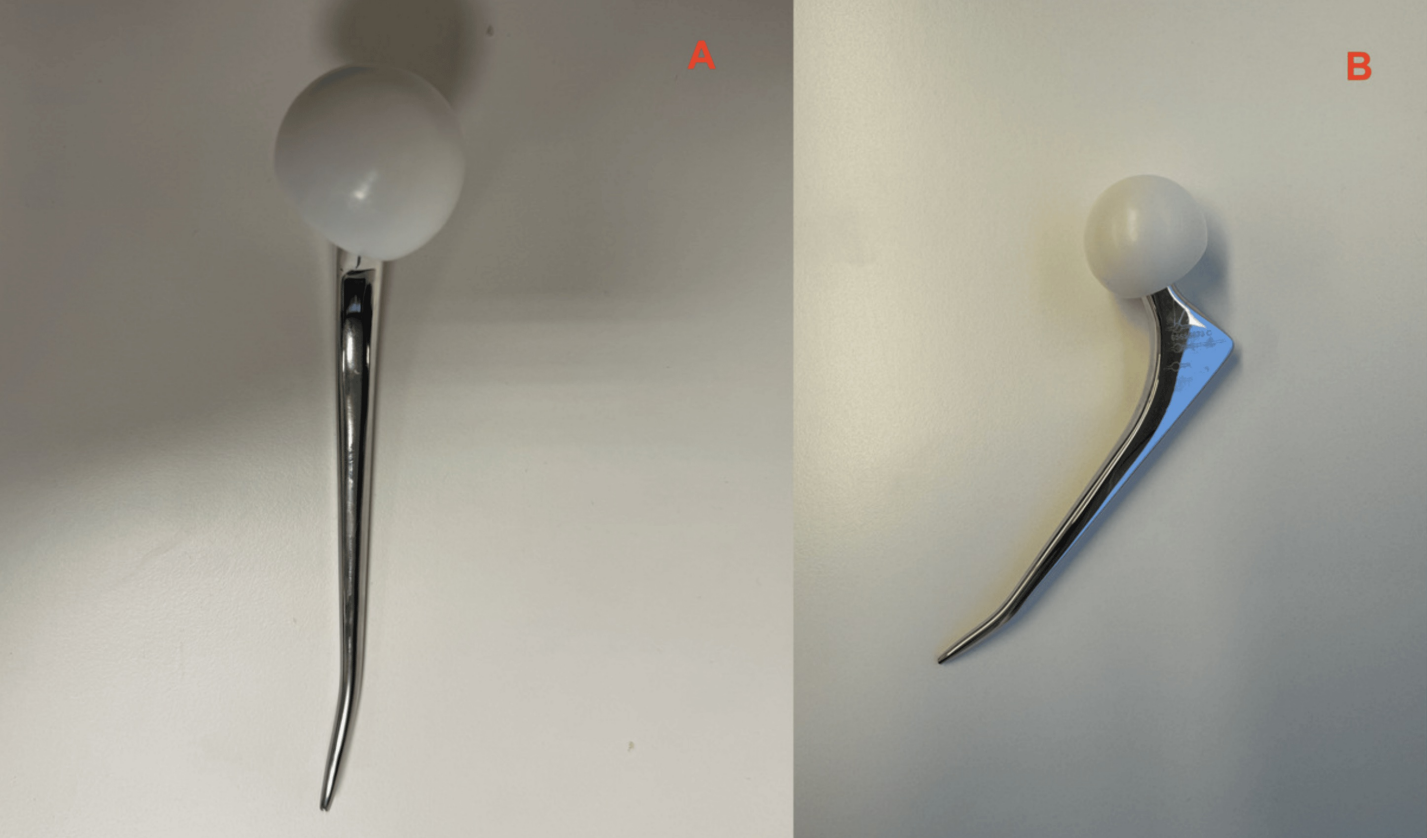
Deformed CPT Stem in vitro Image credits: author. The photos were taken in the OR directly after surgical stem removal. Subsequently, the stem was sent to Zimmer Biomet, the manufacturer, for analysis. 4A: Front view of the CPT Stem in vitro; 4B: Lateral view of the CPT stem in vitro CPT: Collarless, Polished, Double-Taper

The PPF was described as a Vancouver B2 according to the Vancouver femoral fracture classification [5]. The remarkable finding accompanying this fracture was that the distal tip of the femoral CPT stem was deformed and irreversibly bent, without appearing broken (Figure 4). 

A surgical plan was established together with the orthopaedic hip surgeons of the local hospital on how to approach this PPF. Due to the uncommon finding of stem bending, Zimmer Biomet, the manufacturer of the implanted CPT stem, was contacted and available for consultation during the revision surgery. The company had not encountered deformation or bending of their femoral CPT stems before, or in any other of their femoral stem systems.

The material implanted during the primary THA consisted of the commonly used Zimmer Biomet cemented CPT 12/14 size 1 extended femur component, a 22 millimetres cobalt-chromium femoral head and a standard (+0) femoral neck. The acetabular components consisted of a cemented Zimmer Biomet 46 millimetres Avantage cup with 46x22 millimetres acetabular polyethylene (PE) inlay. All implants were cemented using Zimmer Biomet’s vacuum pressurized Optipac^©^ 80 Refobacin^©^ Bone Cement R (Zimmer Biomet, Warsaw, USA). 

While constructing a plan for the revision surgery, the patient's medical history of cognitive impairment and epilepsy resulting in a high risk of falling (again) were important factors to consider. Rigid fixation with a longer femoral stem was preferred to bypass the fracture site. Leaving the original CPT femoral stem in situ was no option at all due to the irreversible deformation. Normally this can be considered in these kinds of fractures since the CPT femoral component is tapered and polished. The retention of the original stem and fracture treatment using open reduction and internal fixation (ORIF) would have been the preferred treatment if the CPT stem had not been deformed. Stem retention provides good long-term results when compared to stem revision and the surgery itself is less intensive for the patient, serving the surgeon and the surgical team [5, 6]. 

The experienced hip surgeons, in consultation with Zimmer Biomet, decided to implant Zimmer Biomet’s 190 millimetres Arcos femoral stem combined with cables or the NCB (Non-Contact Bridging) femoral plate for optimal fixation of the fractured area. The primary THA was fully cemented because of its fracture-related indication (femoral neck fracture), relatively young age of the patient and poor bone quality [7]. Since cementation from the primary THA was only three months prior, removal was expected to be a difficult task. Orthosonics OSCAR3 from Orthofix was available during surgery for optimal cement removal (Orthofix Medical Inc., Lewisville, USA). 

**Figure 5:**
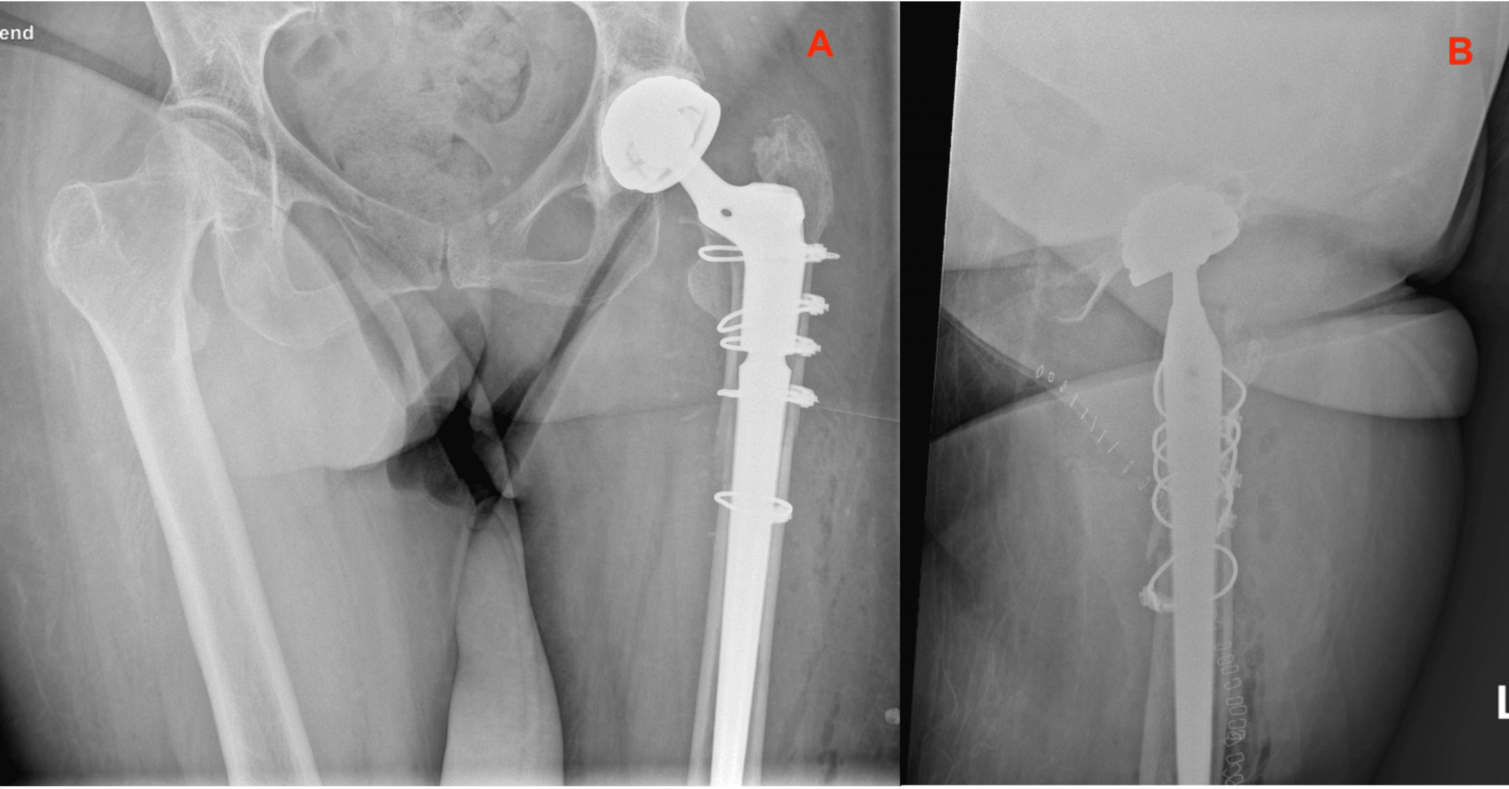
Total Hip Arthroplasty with Arcos stem and cerclages, direct after revision Routine direct postoperative radiographs of the revision Total Hip Arthroplasty with Arcos femoral stem and cerclages (Zimmer Biomet, Warsaw, USA), performed for a Vancouver B2 periprosthetic hip fracture. 5A: Anterior Posterior view of the Pelvis; 5B: Lateral view of the Left Hip

**Figure 6:**
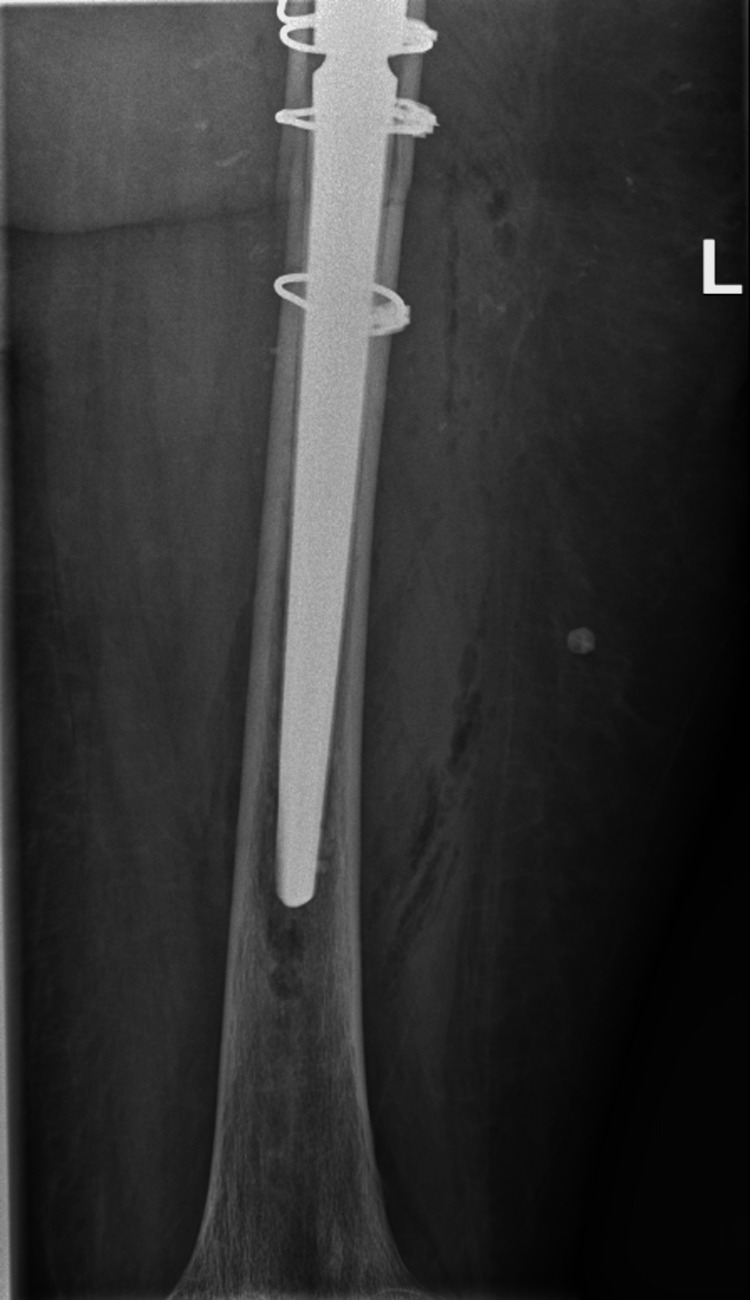
Total Hip Arthroplasty with Arcos stem and cerclages, direct after revision Routine direct postoperative radiographs of the revision Total Hip Arthroplasty with Arcos femoral stem and cerclages (Zimmer Biomet, Warsaw, USA), performed for a Vancouver B2 periprosthetic hip fracture.

Revision was performed using the previous posterolateral incision. The stem was removed, and the cement mantle was evacuated. A 1.8 millimetres cerclage with crimp was placed around the femur to prevent cracks during stem reinsertion. Subsequently, Zimmer Biomet’s uncemented 17x190 millimetres Arcos Splined Tapered Stem and 60 B proximal body were implanted. Additionally, a new Zimmer Biomet 22 millimetres cobalt-chromium femoral head and 46x22 millimetres acetabular PE inlay (Avantage) were mounted. The primary Avantage shell was not revised. After stem placement, the fracture could be fixated using four 1.8 millimetres cerclages with crimp (Cable-Ready® Cable Grip System, Zimmer Biomet) without the need of an NCB femoral plate (Figures 5, 6). 

Rehabilitation after this revision surgery ensued without any complications. The hospital and the patient’s assisted-living facility aimed for a short in-hospital admission since the patient thrived best in her own living environment. During her short postoperative in-hospital stay, mobilization was extended under the supervision of the physiotherapist, the pain gradually diminished, and the wound was healing nicely. Direct postoperative radiographic imaging (Figures 5, 6) demonstrated a well-positioned Zimmer Biomet Arcos femoral revision stem with cerclages around the femoral fracture. There were no signs of new periprosthetic fractures, perioperative complications, or secondary migration of the implants. Two days after the revision, the patient returned to her living facility for further rehabilitation. 

**Figure 7:**
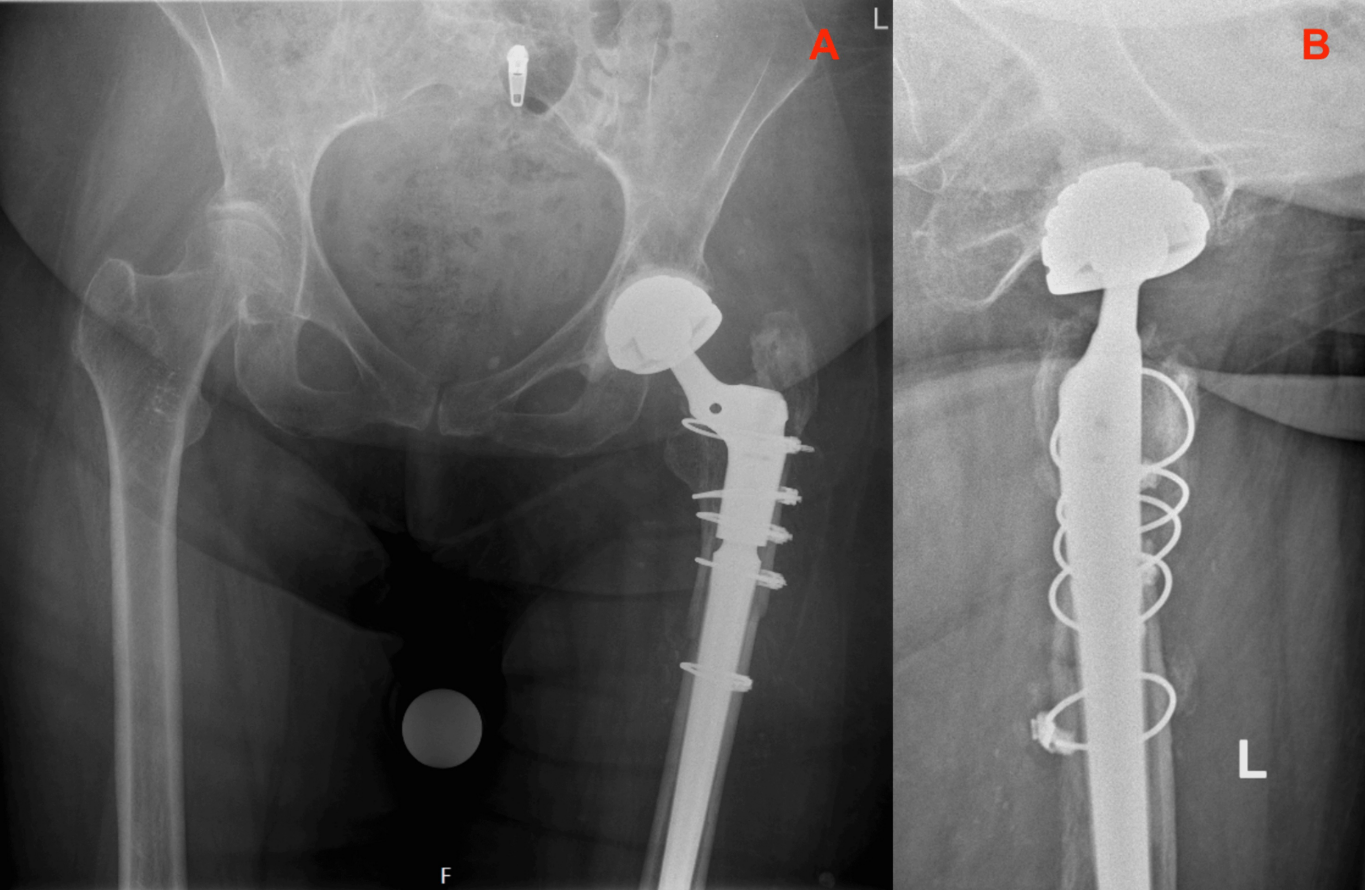
Total Hip Arthroplasty with Arcos stem and cerclages, six months after revision Six months postoperative radiographs of the revision Total Hip Arthroplasty with Arcos femoral stem and cerclages (Zimmer Biomet, Warsaw, USA), performed for a Vancouver B2 periprosthetic hip fracture. 7A: Anterior Posterior view of the Pelvis; 7B: Lateral view of the Left Hip

**Figure 8:**
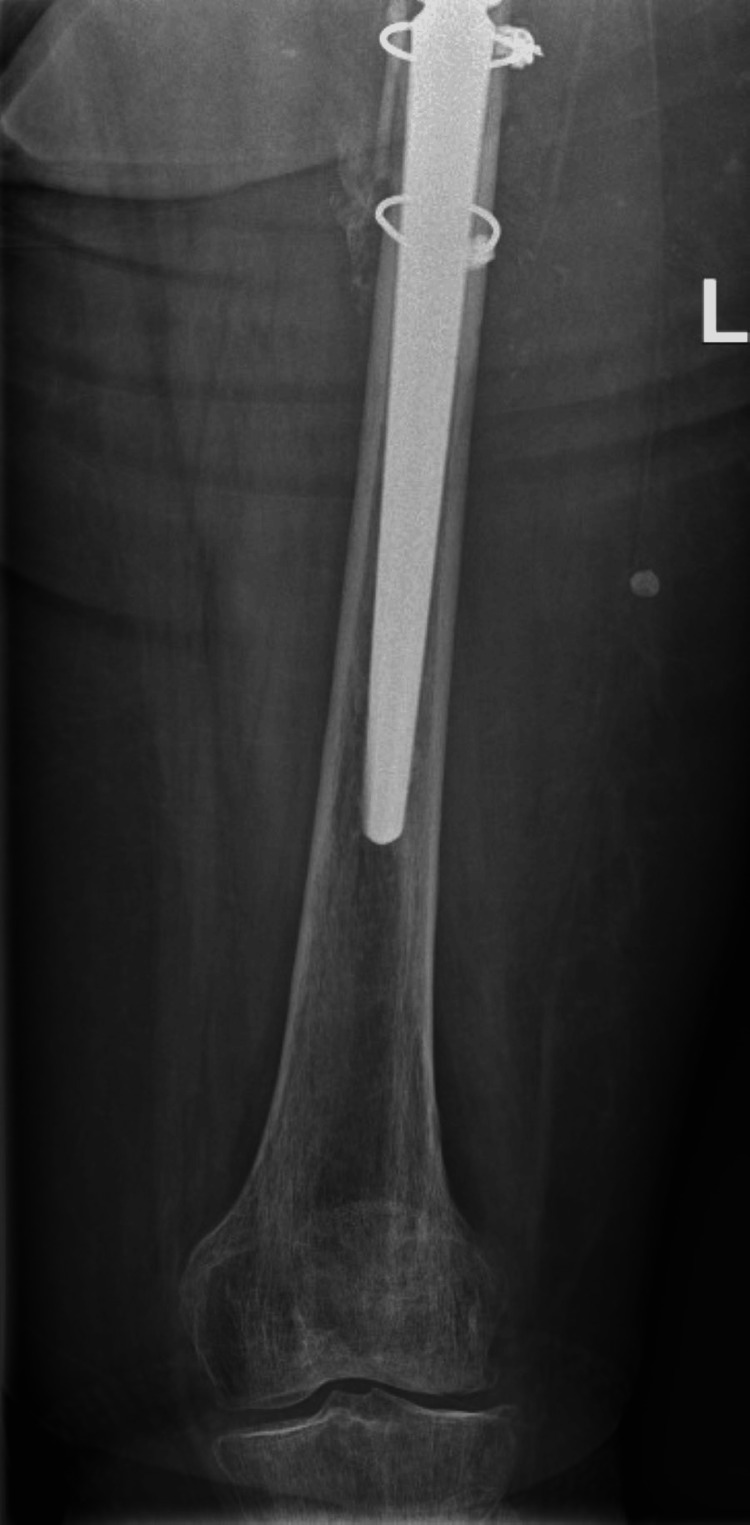
Total Hip Arthroplasty with Arcos stem and cerclages, six months after revision Six months postoperative radiographs of the revision Total Hip Arthroplasty with Arcos femoral stem and cerclages (Zimmer Biomet, Warsaw, USA), performed for a Vancouver B2 periprosthetic hip fracture.

In routine care, patients are followed up two weeks after surgery by phone. Six weeks after surgery they visit the outpatient clinic and radiographic imaging is performed. If there are no problems or abnormalities during these visits, the next and final follow-up is one year after surgery. Because the presented case is remarkable, in combination with the cognitive impairment of this patient, she was closely followed up until six months postoperative. Rehabilitation happened slowly but steadily. Six months postoperative, the patient still used a walking tool during mobilization but without any complaints of pain. Radiographic imaging six months after surgery showed a well positioned Arcos femoral revision stem with cerclages. Progressive consolidation at the site of the previous PPF was visible (Figures 7, 8). With shared decision making, it was decided to further follow up on indication only. 

## Discussion

This case report presents a rare case of women with cognitive impairment and a PPF in THA with the remarkable finding of a CPT femoral stem deformity (bending). Orthopaedic experts and caregivers involved in this case were interested in whether there were any explanations for this uncommon stem deformity. After a thorough case study of the patient’s medical record and the previous THA performed, no signs of complications such as loosening, malpositioning, or lucent lines were visible on radiographic imaging of the left hip and pelvis after the primary THA (Figures 1, 2).

Reflecting on the case, some (perhaps explanatory) remarks on the primary implanted CPT femoral stem could be that it was somewhat undersized (a CPT 12/14 size 2 could have been more appropriate) and the stem was implanted in a slight varus angle (Figure 1). After six weeks, there was a small amount of subsidence (1-2 millimeters) of the stem in the cement mantle, which is a physiological finding related to the design of this taper-slip femoral CPT stem, however, perhaps in retrospect, the cement mantle is slightly inadequate in some places around the stem (Figure 2). 

To better understand this unusual finding of CPT femoral stem bending, a thorough literature study was performed. PPFs after THA are rare but threaded complications. After primary THA the risk of PPF is estimated between 0.4% - 3.5%, with a prevalence of less than 1%. In most cases surgical fixation is indicated. In Vancouver B2 fractures, fixation is often in combination with revision arthroplasty. The fracture is located around or just below the femoral stem in such a manner that the stem is loose [5]. Loosening or a threat of loosening due to the trauma mechanism, extent, and location of the PPF can require (preventive) revision of implants [7]. The surgical plan and strategy for revision is made preoperative based on radiographic imaging, Vancouver classification [5], trauma mechanism and patient specific factors (e.g., age and level of mobility). Based on current postoperative failure rates, loosening of the femoral stem seems to be underdiagnosed. This leads to unexpected intraoperative stem revision or the need for a second revision surgery later when the loosening is suspected on radiographic imaging [8]. 

An infrequent finding in PPFs after THA is the fracturing of implants. Different kinds of femoral and acetabular implant fractures have been described; all are rare [3, 4]. A recent long-term survival analysis of 394 shorter cemented Exeter femoral components (Stryker, Newbury, UK) showed 100% (95%-CI 100 - 100) survival after one-year, 99.5% (95%-CI 96.4 - 99.9) after 10-year, and 92.7% (95%-CI 78.5 - 97.6) after 20-year follow-up [9].

Deformation of implants due to trauma and/or fractures, like in our case, is even rarer. We conducted an extensive search to find literature describing stem deformations of a Zimmer Biomet CPT cemented stem or any kind of polished taper-slip stem designs after THA but found none. 

To dive even deeper into the case, Zimmer Biomet, the manufacturer of the CPT stem, performed a failure analysis. Their technical analysis did not show any manufacturing anomalies, no cracks, damage or fracturing of the particular CPT stem. The material strength, material composition, and dimensional requirements of the particular stem were tested again during this analysis and were all in order. Zimmer Biomet also analyzed the radiographs of the specific patient where the CPT stem was in situ; they did not show any explanations for the deformity either.

CPT stems are made from cobalt-chrome; this yields strength to the point, where plastic deformation occurs prior to a fracture. In our case, the stem bending occurred at an area of the stem with a narrow cross-section distal to the proximal femur. The stem experienced stress within the yield strength but not up to the fracture strength. Zimmer Biomet mentioned that all CPT stems are tested for fatigue strength following ISO and internal standards and all meet the requirements that have been agreed upon with various regulatory bodies.  

Zimmer Biomet stated that if rehabilitation protocols are not followed as prescribed by their Instructions for Use (IFU), it could lead to implant failure that they are not accountable for. Since the CPT stem was implanted in a woman with cognitive impairment, Zimmer Biomet considers it more likely that the rehabilitation instructions are less well understood by this patient than a healthy patient.

In addition to material and manufacture analysis, we asked Zimmer Biomet some more specific questions to understand this rare kind of CPT stem deformation without fracturing. Comparable to current literature, Zimmer Biomet had never seen such a deformation of a CPT stem (or any other of their femoral components) before. They, however, describe that, although uncommon, bending is possible if the incident that led to the bone fracture did not load the stem enough to fracture.

Reflecting on our case, some described risk factors for PPFs can be attributed [10]. Patient-related risk factors were poor bone quality, female gender, and a relatively young age. Although the patient had a low activity level due to her cognitive impairment and epilepsy, the risks of falling and low energetic trauma mechanisms were higher. Her level of understanding due to her severe cognitive impairment is a risk factor during her rehabilitation and mobilization. Controversially, the first surgery was only three months prior, which could be a protective factor due to less implant and cement fatigue providing a more stable environment around the implants. The fact that not only the PPF but also the primary surgical indication was fracture-related partly rejects this protective factor. 

In retrospect, there are some postoperative (bio)mechanical factors which could contribute to the PPF. These include the varus angle of the primary CPT stem and its relatively undersized stem size. In addition, the cement mantle around the primary stem is potentially slightly inadequate - the cement is not completely opaque at the level of the lesser trochanter (Figure 1). These factors could provide unequal force distribution over the femoral implant. Optimal stress distribution in THA is essential to prevent fractures or loosening. This is accomplished by thorough perioperative preparation and measurement of implants [11]. Despite these risk factors, bending of a femoral stem is a very uncommon failure mechanism. 

Current evidence on geometric and mechanical factors contributing to stem deformities is lacking. Published evidence specifically focusing on mechanical factors and higher rates of varus implantation does not support varus angles >5° as a risk factor for complications in THA (including PPFs). Additionally, functional outcomes did not differ with an increased varus angle [12-14]. A bone model study evaluating stem geometry and its relation to PPFs specifically focused on the CPT stem confirmed that stem geometry could not be linked to PPFs [15]. 

Evidence on stem sizes and their relation to PPFs shows more complications using shorter femoral stems, especially in patients with poor bone quality (like in our case). The problem with decreased bone quality is the intraoperative risk of fractures if the stem is too large during implantation [16]. On the other hand, a 15-year follow-up meta-analysis of 2167 Exeter V40 stems (Stryker orthopaedics, Mahwah, USA) found a 0.6% rate of PPFs during the follow-up period [17]. The Exeter stem can be seen as a precursor of the CPT stem since these both are collarless, polished, double-tapered and cemented. 

## Conclusions

To conclude, the patient in our case had a rare PPF after THA with a rare CPT femoral stem deformity. In retrospect, this deformation could possibly be related to some unmodifiable patient-related factors along with some (preventable) surgical or (bio)mechanical risk factors. It is, however, difficult to determine whether and to what extent these risk factors contributed to this uncommon irreversible CPT femoral stem bending in PPF after THA. 

What is informative about this case report for daily orthopaedic practice is that PPFs in THA are dreaded complications. Treatment must be performed and prepared with care and thorough perioperative planning in collaboration with trained colleagues. If stem deformity occurs in PPFs, whether as stem fracture or irreversible bending, it is important to consider patient-related risk factors and the initial stem placement to evaluate the underlying causes of such deformities. In case of doubt, always contact stem manufacturers to aid in the process of understanding why the stem deformity happened. Especially in case of patient-related risk factors, be aware perioperatively that unexpected complications may arise. It is essential to thoroughly discuss potential risks with patients and caregivers, ensuring informed consent is obtained.
